# Silencing of GPNMB by siRNA Inhibits the Formation of Melanosomes in Melanocytes in a MITF-Independent Fashion

**DOI:** 10.1371/journal.pone.0042955

**Published:** 2012-08-13

**Authors:** Ping Zhang, Wei Liu, Cansheng Zhu, Xiaoying Yuan, Dongguang Li, Weijie Gu, Huimin Ma, Xin Xie, Tianwen Gao

**Affiliations:** 1 Department of Dermatology, the General Hospital of the Air Force, Beijing, China; 2 Department of Dermatology, Xijing Hospital, Fourth Military Medical University, Xi'an, China; 3 Shaanxi Provincial Institute for Endemic Disease Control, Xi'an, China; 4 Reg Lab of Tissue Engineering, Faculty of Life Science, Northwest University, Xi'an, China; Institut Jacques Monod, France

## Abstract

**Background:**

Melanosomes are specialized membrane-surrounded organelles, which are involved in the synthesis, storage and transport of melanin. Glycoprotein (transmembrane) non-metastatic melanoma protein b (GPNMB), a melanosome-specific structural protein, shares significant amino acid sequence homology with Pmel-17. Proteomic analysis demonstrated that GPNMB is present in all stages (I-IV) of melanosomes. However, little is known about the role of GPNMB in melanosomes.

**Methodology/Principal Findings:**

Using real-time quantitative PCR, Western blotting and immunofluorescence analysis, we demonstrated that the expression of GPNMB in PIG1 melanocytes was up-regulated by ultraviolet B (UVB) radiation. Transmission electron microscopy analysis showed that the total number of melanosomes in PIG1 melanocytes was sharply reduced by GPNMB-siRNA transfection. Simultaneously, the expression levels of tyrosinase (Tyr), tyrosinase related protein 1 (Trp1), Pmel17/gp100 and ocular albinism type 1 protein (OA1) were all significantly attenuated. But the expression of microphthalmia-associated transcription factor (MITF) was up-regulated. Intriguingly, in GPNMB silenced PIG1 melanocytes, UVB radiation sharply reduced MITF expression.

**Conclusion:**

Our present work revealed that the GPNMB was critical for the formation of melanosomes. And GPNMB expression down-regulation attenuated melanosome formation in a MITF-independent fashion.

## Introduction

The melanosome is a specialized membrane-surrounded organelle, which is involved in the synthesis, storage and transport of melanin. Melanosomes experience four sequential morphological stages (stage I, II, III and IV) during their mature process [1]. Multiple enzymatic and structural proteins are involved in the maturation of melanosomes. To date, 12 proteins have been identified as melanosome-specific proteins, including tyrosinase (Tyr), tyrosinase-related protein 1 (Trp1), tyrosinase-related protein 2 (Trp2, also being known as dopachrome tautomerase), ocular albinism type 1 protein (OA1), melanoma-associated antigen recognized by T cells (MART-1), Pmel17/gp100, vesicle amine transport protein 1 homolog (VAT-1), oculospanin, syntenin, coiled-coil-helix-coiled-coil-helix domain containing 3 (CHCHD3), flotillin-1/2 and glycoprotein (transmembrane) non-metastatic melanoma protein b (GPNMB) [1], [2]. The vital roles of some of these proteins with regards to enzymatic components in melanosome biogenesis are well known, such as Tyr, Trp1 and Trp2 [3], [4]. However, the functions of some structural proteins in the biosynthesis of melanosome are not clear, including the melanosome-specific structural protein GPNMB [1].

GPNMB, a highly glycosylated type I transmembrane protein, was initially cloned from low-metastatic melanoma cells in 1995 [5]. Human GPNMB is comprised of 560 amino acids, which are encoded by a gene localized to chromosome 7p15 in humans [6]. GPNMB shows a high level of structural homology to a well-known melanosomal structural protein, Pmel17 [1], which plays critical roles in the formation of pre-melanosomes [7]. GPNMB consists of several domains including a signal peptide domain (SIG), an N-terminal domain (NTD), a polycystic kidney disease-like domain (PKD), GAP1 (an undefined domain), a kringle-like domain (KRG), and GAP2 (an undefined domain) in the extracellular region, a C-terminal domain (CTD) in the intracellular region and a transmembrane domain (TM) [1]. An arginine-glycine-aspartate (RGD) motif that can bind to integrins [8] may contribute to the melanocyte-keratinocyte adhesion [9]. An isoform of GPNMB has an additional 12 amino acids in the GAP2 domain, probably as a result of alternative splicing. GPNMB expression level in melanocytes was reported to be inversely correlated with the metastatic capacity of human melanomas [5] and linked to the developing retinal pigment epithelium and iris [10]. A premature stop codon mutation in the GPNMB gene was shown to cause iris pigment dispersal in mouse pigmentary glaucoma [11]. Proteomic analysis demonstrated that GPNMB was present in all stages (I-IV) of melanosomes [12], and especially enriched in mature (stage III and IV) melanosomes [1]. All of these results suggest that GPNMB may be important for the formation of melanosomes.

The transcription of GPNMB is regulated by microphthalmia-associated transcription factor (MITF) [13], [14], which belongs to a family of transcription factors that contain a basic helix-loop-helix and leucine-zipper (bHLH-LZ) structure [15]. MITF plays a major role in the transcriptional regulation of more than 25 pigmentation genes, including Tyr, Trp1, Trp2, Pmel17, OA1 and so on [16]. Thus, MITF had been regarded as an essential regulator for melanocyte development, survival and proliferation, too [16].

In the current study, we reported that GPNMB is critical for the formation of early melanosomes and its expression is up-regulated by ultraviolet B radiation (UVB). The expression levels of MITF, Tyr, Trp1, Pmel17 and OA1 were also determined when GPNMB expression was down-regulated by siRNA interference.

## Results

### GPNMB Expression Level was Up-regulated by UVB Radiation

Given that the UVB radiation could up-regulate the expression of Tyr [17], we determined whether the expression of GPNMB could also be modulated by UVB. In a preliminary study, we evaluated the effect of UVB radiation on the viability of cells after 24 hours incubation post-radiation. PIG1 melanocytes exposed to 30 mJ/cm^2^ of UVB radiation showed viability lower than 30%, and melanocytes exposed to 10 mJ/cm^2^ of UVB radiation demonstrated little GPNMB expression change examined by Western blotting (data not shown). While, a dose of 20 mJ/cm^2^ of UVB radiation resulted in (80.84±2.3)% viability and obvious GPNMB expression change (data not shown). So, 20 mJ/cm^2^ was chosen as the optimal UVB radiation dose in the present experiment.

**Figure 1:**
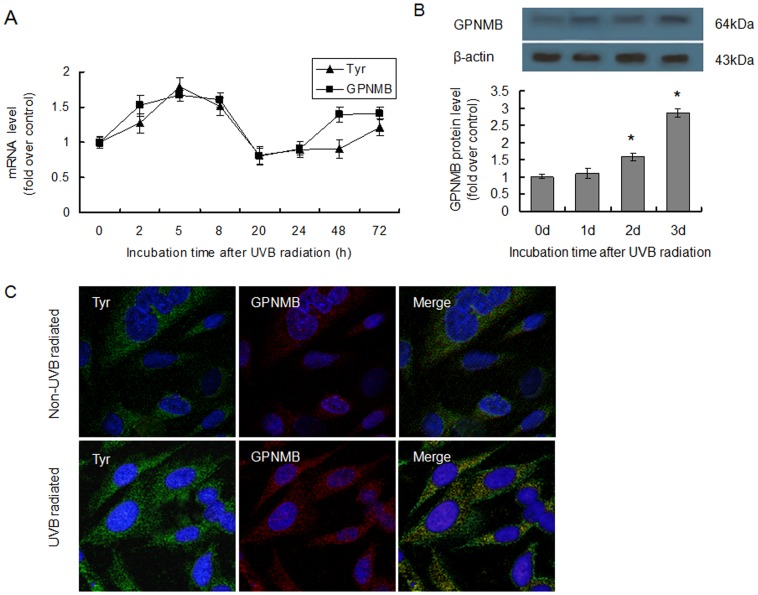
UVB radiation up-regulated the expression of GPNMB. (A) PIG1 melanocytes were exposed to a dose of 20 mJ/cm^2^ UVB radiation and cultured for the indicated times. The mRNA expression levels were determined using real-time quantitative PCR, and were normalized based on the β-actin levels. Data were represented as relative mRNA expression levels, which were the ratio of normalized mRNA expression levels of UVB-irradiated samples to those of non-UVB-irradiated samples. (B) PIG1 melanocytes exposed to a dose of 20 mJ/cm^2^ of UVB radiation were harvested and lysed after the indicated incubation times. Western blotting was performed to examine protein expression of GPNMB. The expression levels of GPNMB were normalized based on the β-actin levels. Densitometric data were represented as mean±SEM in triplicate. *p<0.05. (C) Double immunofluorescence was performed with anti-Tyr antibody (green) to detect melanosomes and anti-GPNMB antibody (red) to identify the expression of GPNMB. Nuclear stained with DAPI (blue) were introduced for visualization purposes. Fluorescence was observed and analyzed with a ﬂuorescence microscope. Left: Tyr staining (in green); Middle: GPNMB staining (in red); Right: merged (in yellow).

Real-time quantitative PCR analysis showed that the transcription of the GPNMB was up-regulated in the first 8 hours, followed by a decrease, and a further up-regulation after 24 hours of incubation (Figure 1A). This variation tendency was, to some extent, in accordance with that of Tyr (Figure 1A), which was known to be regulated by UVB [17]. GPNMB mRNA expression reached a peak value of 1.67-fold more than that of the control at 5 hours (Figure 1A). Using Western blotting, we also showed that the protein expression of GPNMB in the melanocytes could be significantly up-regulated by UVB radiation after 2 days incubation (P<0.05), and the GPNMB proteins kept increasing until the third day at least (Figure 1B). Immunoﬂuorescence analysis also supported the conclusion that the protein expression of GPNMB was up-regulated by UVB radiation, and that GPNMB was enriched within the cytoplasm (Figure 1C). These data made it clear that GPNMB was up-regulated by UVB radiation.

### GPNMB-siRNA Transfection Suppressed GPNMB Expression Efficiency

**Figure 2:**
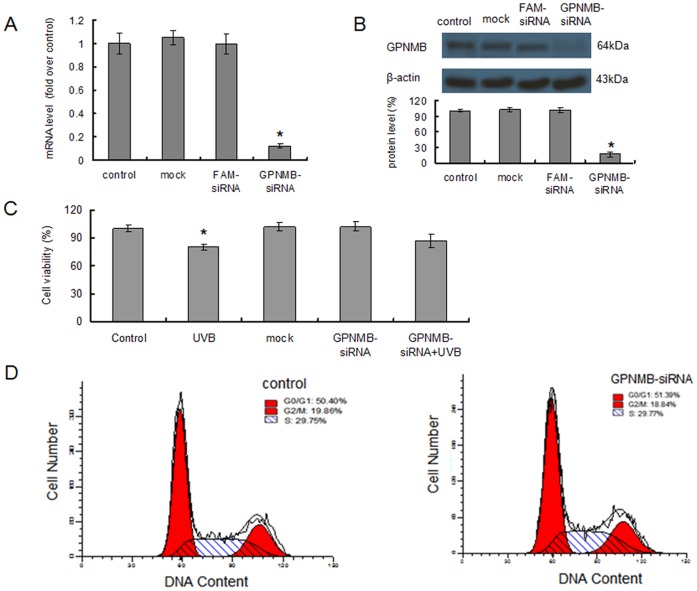
The GPNMB-siRNA transfection reduced GPNMB expression but didn’t affect cell viability. The PIG1 melanocytes were untreated, transfected with FAM-siRNA, or transfected with GPNMB-siRNA, respectively. (A) After 6 hours incubation, real-time quantitative PCR was performed to determine the mRNA alteration of GPNMB. β-actin was used as reference. (B) After 72 hours incubation, protein levels of GPNMB were determined by Western blotting with specific Ab. β-actin was used as reference. GPNMB expression was selectively and effectively inhibited by GPNMB-siRNA transfection. (C) After different treatment, viability of PIG1 melanocytes was assessed using a CellTiter-Blue® Cell Viability Assay Kit. Single UVB radiation inhibited cell viability significantly. While, single GPNMB-siRNA or both GPNMB-siRNA and UVB treated had little affect on cell viability. (D) After 24 hours incubation, cell cycle was determined by flow cytometry basing on propidium iodide (PI) staining. *p<0.05 by Student's *t* test when compared with the control group.

To examine whether GPNMB-siRNA could selectively knock down GPNMB gene expression, PIG1 melanocytes were transfected with either GPNMB-siRNA or a negative control, and the change of GPNMB mRNA and protein levels were examined by Real-time quantitative PCR and Western blotting, respectively. As shown in Figure 2A, GPNMB-siRNA treatment led to an obvious decline of GPNMB mRNA level compared to non-treatment control. Protein level of GPNMB was also significantly decreased in the GPNMB-treatment group (Figure 2B). But the protein decrease lagged behind the alteration of GPNMB mRNA. These results indicated that GPNMB-siRNA transfection was effective.

Cell viability assay demonstrated that single UVB radiation significantly inhibited cell viability (P<0.05) as expected (Figure 2C). While, a single GPNMB-siRNA or both GPNMB-siRNA and UVB treated showed little affect on cell viability (Figure 2C). But the cell viability caused by UVB exposure was not statistically different between the naive and the GPNMB silenced melanocytes (Figure 2C). This indicated that GPNMB-siRNA transfection didn’t affect viability of melanocytes PIG1, even after UVB irradiation. The cell cycle was also not affected by GPNMB-siRNA treatment (Figure 2D).

### GPNMB Knockdown Reduced the Formation of Melanosomes

**Figure 3:**
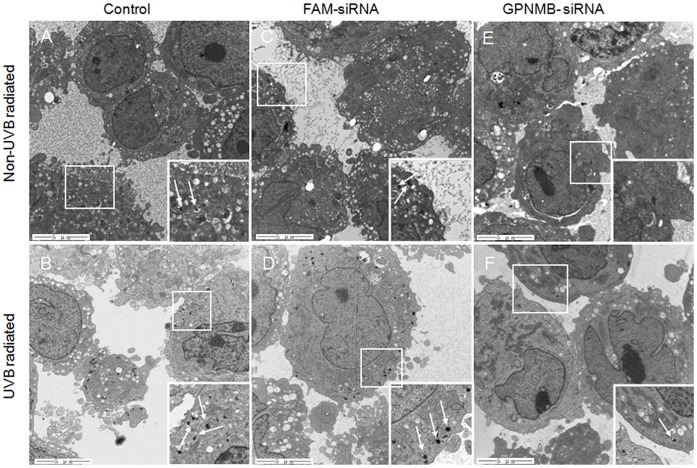
TEM analysis of PIG1 melanocytes with different levels of GPNMB expression modulated by different treatments. PIG1 melanocytes cultured on 35 mm dishes were treated with UVB radiation, siRNA or both. After 72 hours of incubation, cells were harvested for TEM analysis. UVB radiation increased the number of mature melanosomes. While, GPNMB-siRNA treatment led to a sharply decrease of the number of melanosomes in all stage. Inset showed a 2× magnification of the indicated region of the cell. Arrows indicated mature melanosomes.

Aiming to demonstrate the relationship between GPNMB expression and melanosome formation, PIG1 melanocytes with different levels of GPNMB expression modulated by methods of UVB radiation or RNA interference were analyzed by TEM. The naïve PIG1 melanocytes demonstrated numerous melanosomes in stage I or stage II, and sparse melanosomes in stage IV (Figure 3A). Following UVB radiation, a noticeable change was the boost of mature melanosomes (stage IV), although the early melanosomes (stage I and stage II) were still abundant (Figure 3B). Transfection with negative control FAM-siRNA led to little change in the number of melanosomes for either UVB treated or untreated melanocytes as expected (Figure 3D and C). However, when GPNMB expression was knocked down by GPNMB-siRNA transfection, we observed few melanosomes (including stage I and stage II) in the vast majority of melanocytes (Figure 3E). This result indicated that GPNMB contributed mainly to the formation of the early melanosomes. Although it was reported previously that UVB radiation could enhance melanosome formation, UVB radiation failed to promote melanosome formation in GPNMB-siRNA transfected melanocytes (Figure 3F). Taken together, these results suggest that GPNMB is critical for the formation of early melanosomes.

### Tyr Expression was Attenuated by GPNMB-siRNA Transfection

**Figure 4:**
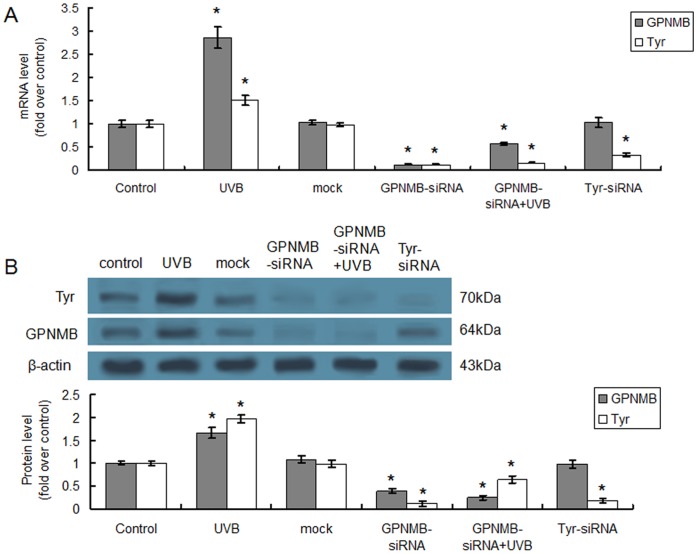
Tyr expression was decreased by GPNMB-siRNA transfection. The mRNA (A) or protein (B) expression levels of Tyr and GPNMB in melanocytes PIG1 treated by UVB radiation, GPNMB-siRNA transfection or both of them were determined using real-time quantitative PCR or Western blotting, respectively. The effect of Tyr-siRNA treatment on GPNMB and Tyr expression levels were also determined using real-time quantitative PCR and Western blotting. Data were normalized based on the β-actin levels and were represented as relative expression levels. *p<0.05 by Student's *t* test when compared with the control group.

Since the vital role of Tyr in melanin synthesis, we asked whether Tyr expression was influenced by GPNMB knockdown. Real-time quantitative PCR analysis demonstrated that the transcription of the Tyr was significantly restrained by GPNMB-siRNA transfection (P<0.05), even with an additional UVB radiation (Figure 4A). Protein expression of Tyr was also suppressed obviously (P<0.05) by GPNMB-siRNA transfection, but this suppression could be partly alleviated by additional UVB radiation (Figure 4B).

To verify the interaction between GPNMB and Tyr, Tyr was also knocked down by siRNA targeting Tyr. As shown in Figure 4A and B, Tyr-siRNA treatment led to a significant decline of Tyr mRNA and protein levels, whereas the expression of GPNMB was unaltered, indicating there was no interaction between GPNMB and Tyr.

### GPNMB Knockdown Reduced Melanosome Formation in a MITF-independent Fashion

**Figure 5:**
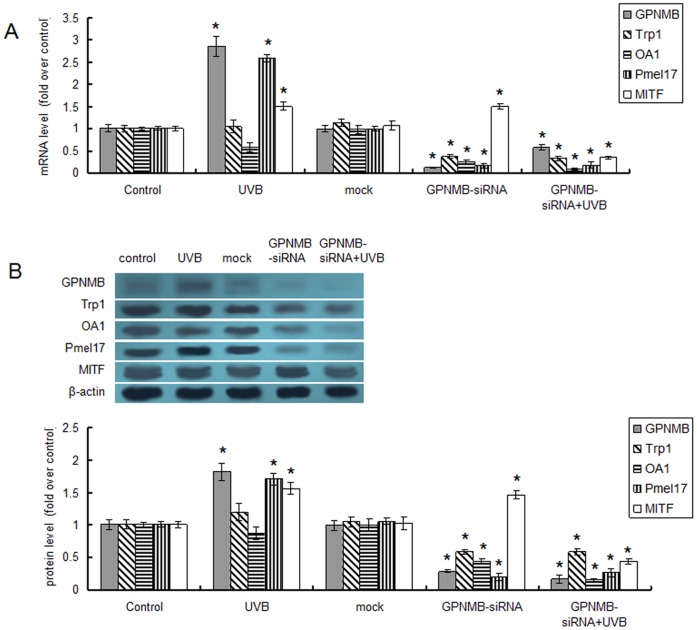
The mRNA and protein expression levels of Tyr, Trp1, Pmel17, OA1 and MITF were modulated by GPNMB down-regulation. The mRNA (A) and protein (B) expression levels were evaluated using real-time quantitative PCR and Western blotting, respectively. Data were normalized based on the β-actin levels and were represented as relative expression levels. *p<0.05 by Student's *t* test when compared with the control group.

Apart from Tyr, other melanosomal proteins such as Trp1, Pmel17 and OA1 were also reported to be important for melanosome formation. To reveal the mechanism by which GPNMB involved in melanosome formation, we also investigated the effect of GPNMB knockdown on these proteins. In the present study, GPNMB-siRNA transfection also significantly suppressed the mRNA and protein expression of all these proteins (Figure 5A and B). And this suppression failed to be alleviated by UVB radiation (Figure 5A and B).

Of note, all these proteins were transcriptionally regulated by MITF, a central regulator in melanocytes [16]. We speculated that the GPNMB-siRNA transfection might attenuate melanosome formation via reducing the expression of MITF. Real-time quantitative PCR and western blotting were performed to evaluate the expression level of MITF. As shown in Figure 5A and B, single UVB radiation up-regulated the mRNA and protein expression of MITF, which was in accordance with the finding of Mizutani [18]. The expression of MITF didn’t decrease but increased after GPNMB-siRNA transfection (Figure 5). Hence, the silencing of GPNMB expression attenuated melanosome formation in a MITF-independent manner. Surprisingly, UVB radiation didn’t lead to higher MITF expression but sharply reduced MITF expression in GPNMB silenced PIG1 melanocytes (Figure 5A and B). Other unknown signal pathways might be involved in the role of GPNMB in the signaling pathway induced by UVB irradiation.

## Discussion

Melanosomes experience four sequential morphological steps as they mature [1]. Stage I melanosomes are round, membrane-surrounded, and electron-lucent vesicles that are generally found in the perinuclear area. Stage II melanosomes are characterized by the elongation of the vesicle and the appearance of distinct fibrillar structures. Melanin synthesis begins and the pigment is deposited uniformly on the internal fibrils, at which time the organelles are termed stage III melanosomes. In highly pigmented tissues, melanin synthesis and deposition continues until little or no internal structures are visible, at which time they are termed stage IV melanosomes. Previously works have showed that GPNMB is expressed in all stages of melanomas or melanocytes. In SK-MEL-28 melanomas, which contain only stage I and II melanosomes [19], and WM266-4 melanomas, which contain only stage I melanosomes, GPNMB is expressed normally [20]. Tomihari and colleagues [9] showed that GPNMB expression was up-regulated by UVA radiation. While in our current study, GPNMB expression was up-regulated by UVB radiation in melanocyte cell line PIG1, too. And the up-regulation of GPNMB by UVB radiation in melanocyte cell line PIG1 almost mimicked those for Tyr. Given that GPNMB and Tyr were both transcriptionally regulated by MITF [16] and the expression of MITF was up-regulated by UVB irradiation [18], we speculated that UVB exposure increased the expression of GPNMB by up-regulating the expression of MITF.

After UVB radiation, we observed an increased number of mature melanosomes in melanocyte cell line PIG1 (Figure 3B). The appearance of mature melanosomes may be as a result of the promoting effect of UVB on melanin synthesis, and, in turn, the maturation of melanosomes. Multiple melanosomal genes modulated by UVB might contribute to the maturation of melanosomes [21], such as *MITF* and its target genes. *GPNMB* as one of the target genes of MITF might be involved in this event.

Previous studies have demonstrated various roles of GPNMB, as it expressed in a diverse range of cells [22], [23], [24]. However, the function of GPNMB in the formation of melanosomes had not yet been fully understood. Pmel17, a melanosome-specific structural protein, was shown to initiate pre-melanosome morphogenesis within multivesicular bodies [7], and was required for the formation of melanosomal matrix for melanin deposition [25]. Therefore, stage II melanosomes could not form if Pmel17 was not processed correctly, since the maturation of Pmel17 underwent cleavage three times [25] and MART-1 was indispensable for Pmel17 function [20]. Because the melanosome-specific structural protein GPNMB structurally resembles Pmel-17, it is reasonable to believe that GPNMB also plays an important role in melanosome. Using siRNA interference, we revealed firstly that the knockdown of GPNMB expression led to a sharply decreased number of melanosomes in melanocyte cell line PIG1, suggesting that GPNMB was critical for melanosome formation. However, the expression of some melanosome associated proteins such as Tyr, Trp1, Pmel17 and OA1 were all inhibited following GPNMB silencing in the present study. We also generated primary melanocytes from teenaged foreskin according to the normal method [26] and obtained the similar results using these primary melanocytes in all the tests in our study (data not shown). To assay the protein expression profile after GPNMB silencing, we had tried to isolate pure melanosomes. But the melanosomes were not successfully isolated because of their limited numbers in GPNMB silenced melanocytes (data not shown). Whether GPNMB silencing inhibited melanosome formation in a direct way or an indirect way via inhibiting these melanosome associated proteins remained to be elucidated. We supposed that there were two possibilities to interpret the roles of GPNMB in melanosome formation: 1) GPNMB was an indispensible structural component in melanosomes. In this case, the decrease of Tyr, Trp1, Pmel17 and OA1 were more likely to be a simple consequence of melanosome decline. Using transmission electron microscopy, Haraszti [27] demonstrated that the phenotypes of iridial melanosomes in GPNMB mutant mice were identical to that of wild type. But synchrotron-based X-ray absorption near-edge structure analysis revealed multiple spectral phenotypes [27]. These indicated GPNMB didn’t influence the ultrastructure but the chemical composition of mice iris melanosomes. Mouse and human GPNMB share a 71% amino acid sequence homology, and both contain the 8 domains described above [9]. However, mouse GPNMB contains 11 N-glycosylation sites, which is one fewer than that of human GPNMB [9]. If GPNMB was an indispensible structural component in melanosomes, it was likely that GPNMB might play different roles in human and mouse melanocytes. The additional N-glycosylation site might mainly contribute to this difference. 2) The other possibility was that GPNMB knockdown disturbed the normal signaling pathways involved in the expression of melanosomal proteins. If so, the decrease of melanosomes after GPNMB-siRNA transfection might be a result of the down-regulation of these important melanosomal proteins. Structural analysis of GPNMB demonstrated that the intracellular domain of GPNMB contained an immunoreceptor tyrosine-based activation motif (ITAM)-like motif (YxxI) [28]. In fact, Tomihari [9] had showed that cross-linking of GPNMB by monoclonal antibodies induced tyrosine phosphorylation in dendritic cells. Thus, it was not impossible that GPNMB was involved in some related signaling pathways.

MITF was considered to play a central role in the regulation of melanocyte development, survival and proliferation [16]. More than 25 pigmental genes were targets of MITF [16], [29]. It was well known that α-melanocyte-stimulating hormone (α-MSH) activated its downstream signal transducer MITF to stimulate gene expression was the primary mechanism in melanin synthesis. In the present study, GPNMB knockdown decreased the expression of the target genes of MITF such as Tyr, Trp1, Pmel17 and OA1, but led to an enhancement of MITF expression. Thus, the melanosome formation inhibited by GPNMB knockdown was MITF-independent. We speculated that the upstream pathway involved in MITF transcription was influenced by GPNMB knockdown. The regulatory mechanism MITF expression was very complex. MITF contained a strong transcription activation domain (TAD) placed at its N-terminal and a weak TAD placed at its C-terminal [30]. At least, four transcription factors had been proven to be involved in the transactivation of the MITF gene in melanocytes, including the paired box-containing transcription factor PAX3, a sex determining region Y (SRY) family member SOX10, the Wnt/β-catenin pathway effector LEF-1 and the cAMP pathway effector cAMP response element binding (CREB) [16]. In addition, the cut-homeo-domain transcription factor Onecut-2 and peroxisome proliferator-activated receptor γ (PPARγ) were also reported to stimulate MITF promoter [31], [32]. Breast cancer suppressor candidate-1 (BCSC-1) could down-regulate MITF by binding to SOX10 [33]. MITF expression could also be inhibited by transcription factor GL12 and transforming growth factor-β [34]. In melanoma cells, the POU domain bearing brain-2/POU class 3 homeobox transcription factor 2 (Brn2) repressed MITF expression [35]. The Wnt/β-catenin pathway had also been implicated in the regulation of MITF expression [15]. This signaling pathway is triggered by the binding of secreted Wnt growth factor proteins to Frizzled/low-density lipoprotein receptor-related protein receptor complexes. Then, the degradation of β-catenin is blocked. This leads to the accumulation of β-catenin and subsequent nuclear translocation, where it heterodimerizes with the members of the T-cell factor (TCF/LEF) family. The β-catenin/LEF complexes can lead to the activation of specific target genes such as MITF [36]. MITF could also function as a non-DNA-binding co-factor for LEF-1 [15] and interact directly with β-catenin [36]. Therefore, a feedback loop is supposed to control the activity of this pathway. Taken together, the expression of MITF is regulated by a complex network. GPNMB might be involved in the network in a novel unknown mechanism.

In conclusion, this report showed that the silencing of GPNMB by siRNA inhibited the formation of melanosomes in melanocyte cell line PIG1 in a MITF-independent fashion. Our findings suggested that GPNMB might be a promising target for pigmentary disorders treatment.

## Materials and Methods

### Antibodies

Rabbit anti-GPNMB polyclonal antibody (Ab), rabbit anti-Tyr polyclonal Ab, rabbit anti-Pmel17 polyclonal Ab, rabbit anti-OA1 polyclonal Ab, rabbit anti-Trp1 polyclonal Ab, rabbit anti-MITF polyclonal Ab, rabbit anti-β-actin polyclonal Ab and horseradish peroxidase (HRP)-conjugated goat anti-rabbit immunoglobulin G (IgG) were purchased from Abcam (Cambridge, MA). Mouse monoclonal Ab to Tyr was purchased from Abgent (San Diego, CA). Phycoerythrin (PE)-conjugated goat anti-rabbit IgG and Fluorescein isothiocyanate (FITC)-conjugated rabbit anti-mouse IgG were purchased from Invitrogen (Carlsbad, CA).

### Cell Lines and Cell Culture Conditions

The immortalized human melanocyte cell line PIG1 was a gift from Professor Caroline Le Poole [26] from the Department of Dermatology, University of Cincinnati, USA. Cryopreserved cells were rapidly thawed in a 40°C water bath and then cultured in 35 mm petri-dishes. The medium used was 254 medium supplemented with 5% fetal calf serum (FCS) and human melanocyte growth supplement (S-002-5), and samples were incubated at 37°C in a humidified atmosphere with 5% CO_2_. Four hours later, the supernatant was removed and fresh medium was added for continued culturing. After reaching 80% confluence, cells were sub-cultured by trypsinization and suspended using medium 254 supplemented with S-002-5 and 5% FCS. Cells were then dispensed and incubated according to each experimental design. The medium 254, FCS and S-002-5 were all purchased from GIBCO BRL (Gaithersburg, MD).

### Cell Viability Assay

Cell viability assay was performed using a CellTiter-Blue® Cell Viability Assay Kit (Promega, Madison, Wisconsin) according to the manufacture introduction.

### Cell Cycle Assay

Cell cycle assay was performed according to the method published previously [37]. Briefly, cells were washed with PBS, harvested by trypsinization, fixed in 70% ethanol, and then labeled with propidium iodide (PI) by incubation for 30 min at room temperature in PBS containing 50 µg/ml PI and 1 mg/ml ribonuclease A. The DNA content per nucleus was analyzed by a FACScan flow cytometer (Becton Dickinson, San Jose, CA).

### UVB Radiation

UVB radiation was performed using a modification of a previously published protocol [38]. To summarize, PIG1 melanocytes were cultured in 35 mm petri-dishes, and were washed once with phosphate-buffered saline (PBS). Cells were then covered with 1 ml PBS and exposed to a UVB (wavelength 296–298 nm) dose range between 0 and 30 mJ/cm^2^ in order to determine the optimal UVB dose. The UVB was emitted by SS-01B-2 UV phototherapy equipment (Shanghai Sigma High Technology Co., LTD, Shanghai, China) with a light intensity of 11.2 mW/cm^2^. Immediately after radiation, the PBS was replaced by 2 ml fresh complete medium, and cells were further cultured for a period before being harvested.

### RNA Extraction and Real-time Quantitative PCR

Total RNA was extracted using RNAiso plus kit (Takara, Dalian, China).The cDNA was synthesized by reverse transcription of total RNA, using the PrimeScript® RT reagent kit (Takara, Dalian, China) with oligo-dT primers, based on the manufacturer’s instructions. Real-time quantitative PCR reactions were performed on the Bio-Rad iQ5 real-time thermal cyclers using SYBR® *Premix Ex Taq*™ II kit (Takara, Dalian, China). The real-time quantitative PCR reaction system and cycling parameters were based on the thermal cycler dice® real time system. 25 µl of amplification mixture was used, which contained 20 ng cDNA and 0.4 µM primers specific for GPNMB (sense: 5′-AAGTGAAAGATGTGTACGTGGTAACAG-3′, anti-sense: 5′-TCGGATGAATTTCGATCGTTCT-3′), Tyr (sense: 5′-GGCCTCAATTTCCCTTCACA-3′, anti-sense: 5′-CAGAGCACTGGCAGGTCCTAT-3′), MITF (sense: 5'-TTGATGGATCCGGCCTTGCAAATG-3', anti-sense: 5'-TATGTTGGGAAGGTTGGCTGGACA-3'), Trp1 [39] (sense: 5'-ATACTGGGACCAGATGGCAACACA-3', anti-sense: 5'-AAGCGGGTCCTTCGTGAGAGAAAT-3'), Pmel17 (sense: 5'-CCCCAGGAAACTGACGATGC-3', anti-sense: 5'-AGCCACAGGAGGTGAGAGGAAT-3'), OA1 (sense: 5'-TCCTCTCGTTCTCCGTGTA-3', anti-sense: 5'-CTGGTGGCTGTTTTGCTAT-3'), or β-actin (sense: 5′-CTGGAACGGTGAAGGTGACA-3′, anti-sense: 5′-AAGGGACTTCCTGTAACAATGCA-3′). These primers were all synthesized by Sangon Biotech Co., Ltd. (Shanghai, China). The cycling conditions were polymerase activation for 30 s at 95°C, 40 cycles of amplification each consisting of 95°C for 5 s, 60°C for 20 s, and 1 cycle of dissociation consisting of 95°C for 15 s, 60°C for 30 s, and 95°C for 15 s. All reactions were performed in triplicate, and results were represented as relative mRNA expression data calculated according to the 2^−ΔΔC^_T_ method [40]. This method involved mRNA expression levels being normalized based on β-actin levels. Relative mRNA expression levels were the ratio of normalized mRNA expression levels of treatment group to control group.

### Western Blotting

Total protein extracts of PIG1 melanocytes were prepared using RIPA lysis buffer (Beyotime, Nantong, China) according to the operating instructions. The protein concentration in the lysates was evaluated using a BCA protein assay kit (Beyotime, Nantong, China). For Western blotting, 40 µg proteins in the lysates were separated on an SDS-polyacrylamide gel, and the fractionated proteins were then transferred from the gel onto the nitrocellulose membrane (Pharmacia, Piscataway, NJ) in a semi-dry trans-blot apparatus. The nitrocellulose membrane was blocked with 10% defatted milk in PBS at 4°C overnight, and then incubated with the primary Ab diluted in defatted milk for 60 min. This was followed by four washes with TBST buffer (0.05 mol/l Tris, 0.15 mol/l NaCl, 0.05% Tween 20). Later, the blots were incubated with HRP-conjugated second antibody for 60 min at 37°C, and washed for a further four times in TBST buffer, followed by detection using ECL reagents (Boehringer Mannheim, Mannheim, Germany) according to the manufacturer’s instructions, and exposure to photographic film (Kodak, Rochester, NY). All experiments were performed in triplicate, and results were normalized according to β-actin.

### Immunofluorescence Microscopy

A total of 3×10^5^ PIG1 melanocytes were plated onto a glass coverslip and cultured overnight. After UVB radiation and 3 days incubation, cells were washed twice with PBS and fixed with methanol at −20°C for 15 min, followed by two further washes with PBS. Coverslips were incubated with primary Ab (rabbit anti-GPNMB polyclonal Ab or mouse anti-Tyr monoclonal Ab) at 4°C for 1 hour, followed by being washed four times with PBS. PE-conjugated goat anti-rabbit IgG and FITC-conjugated rabbit anti-mouse IgG were used as secondary Ab. At the end of treatment, the cells were incubated with 1 µg/ml fluorescent dye DAPI (Sigma-Aldrich, Saint Louis, MO) and PBS for 30 min to evaluate the nuclear position. Images of treated cells were captured using an Olympus FluoView FV1000 confocal laser scanning microscope (Olympus, Tokyo, Japan) and analyzed with Olympus FV1000 software FV10-ASW version 2.1b.

### siRNA Transfection

For targeted knockdown of GPNMB, a mixture of four pairs of GPNMB-siRNA were designed according to a human GPNMB gene transcript (NCBI GenBank accession number, NM_002510) and synthesized by Genetimes Technology (Shanghai, China). Their nucleotide sequences were GPNMB-homo-248∶5′-GGGCAAUGAAAGACCUUCUTT-3′ (sense) and 5′-AGAAGGUCUUUCAUUGCCCTT-3′ (antisense), GPNMB-homo-429∶5′-GUGGGCUCAAAUAUAACAUTT-3′ (sense) and 5′-AUGUUAUAUUUGAGCCCACTT-3′ (antisense), GPNMB-homo-1228∶5′-CUGCCAGAUUACAGAUAUTT-3′ (sense) and 5′-AUAUCUGUUAACUGGCAGTT-3′ (antisense), and GPNMB-homo-1345∶5′-GCUCCCUAAUAGACUUUGUTT-3′ (sense) and 5′-ACAAAGUCUAUUAGGGAGCTT-3′ (antisense), respectively. A pair of FAM-siRNA with nucleotide sequences of 5′-UUCUCCGAACGUGUCACGUTT-3′ (sense) and 5′-ACGUGACACGUUCGCAGAATT-3′ (antisense) was used as negative control. The Tyr-siRNA with nucleotide sequences of 5′-GUCUCCUCUAAGAACCUGA-3′ (sense) and 5′-UCAGGUUCUUAGAGGAGAC-3′ (anti-sense) were used to targeted knock down of Tyr [41]. For transfection, 5×10^4^ PIG1 melanocytes were seeded in each cell of 24-well micro-plates, grown for 1 day to reach 30–50% confluence, and incubated with a mixture of 6 pmol siRNA and 1 µl Lipofectamine™ RNAiMAX (Invitrogen, Carlsbad, CA) in 100 µl serum-free medium 254 at 37°C with 5% CO_2_. Twenty four hours later, the transfection efficiency was examined by Western blotting as described above.

### Transmission Electron Microscopy

For TEM analysis, PIG1 melanocytes were fixed with 2% glutaraldehyde in Ca^2+^ and Mg^2+^ free Dulbecco’s phosphate-buffered saline at 4°C for 2 hours, followed by two washes with D-PBS. Then, the cells were post-fixed with 1% osmium tetroxide at 4°C for 2.5 hours. After fixation, they were dehydrated in a graded series of ethanol and acetone at 4°C, and embedded in epoxy resin at room temperature. Three hours later, cells were solidified for 48 hours at 60°C. Ultrathin sections were obtained using a RMC-MT6000XL ultra-microtome and stained with uranyl acetate and lead citrate. Samples were then examined under an electron microscope (JEM-1200EX, JEOL, Japan) at an accelerating voltage of 75 kV.

### Statistical Analysis

Data are expressed as mean ± SEM from a minimum of three experiments. Student’s *t* test was used to determine the significance of differences in multiple comparisons. A value of p<0.05 was regarded as statistically significant.

## References

[1] Hoashi T, Sato S, Yamaguchi Y, Passeron T, Tamaki K, et al (2010) Glycoprotein nonmetastatic melanoma protein b, a melanocytic cell marker, is a melanosome-specific and proteolytically released protein. FASEB J 24: 1616–1629. 20056711 10.1096/fj.09-151019 PMC2879953

[2] Basrur V, Yang F, Kushimoto T, Higashimoto Y, Yasumoto K, et al (2003) Proteomic analysis of early melanosomes: identification of novel melanosomal proteins. J Proteome Res 2: 69–79. 12643545 10.1021/pr025562r

[3] Kim HJ, An SM, Boo YC (2008) A dual mechanism of 4-hydroxy-5-methyl-3[2H]-furanone inhibiting cellular melanogenesis. J Cosmet Sci 59: 117–125. 18408869

[4] Olivares C, Solano F (2009) New insights into the active site structure and catalytic mechanism of tyrosinase and its related proteins. Pigment Cell Melanoma Res 22: 750–760. 19735457 10.1111/j.1755-148X.2009.00636.x

[5] Weterman MA, Ajubi N, van Dinter IM, Degen WG, van Muijen GN, et al (1995) nmb, a novel gene, is expressed in low-metastatic human melanoma cell lines and xenografts. Int J Cancer 60: 73–81. 7814155 10.1002/ijc.2910600111

[6] Kuan CT, Wakiya K, Dowell JM, Herndon JE 2nd, Reardon DA, et al (2006) Glycoprotein nonmetastatic melanoma protein B, a potential molecular therapeutic target in patients with glioblastoma multiforme. Clin Cancer Res 12: 1970–1982. 16609006 10.1158/1078-0432.CCR-05-2797

[7] Berson JF, Harper DC, Tenza D, Raposo G, Marks MS (2001) Pmel17 initiates premelanosome morphogenesis within multivesicular bodies. Mol Biol Cell 12: 3451–3464. 11694580 10.1091/mbc.12.11.3451 PMC60267

[8] Ruoslahti E (1996) RGD and other recognition sequences for integrins. Annu Rev Cell Dev Biol 12: 697–715. 8970741 10.1146/annurev.cellbio.12.1.697

[9] Tomihari M, Hwang SH, Chung JS, Cruz PD Jr, Ariizumi K (2009) Gpnmb is a melanosome-associated glycoprotein that contributes to melanocyte/keratinocyte adhesion in a RGD-dependent fashion. Exp Dermatol 18: 586–595. 19320736 10.1111/j.1600-0625.2008.00830.x PMC2774115

[10] Bachner D, Schroder D, Gross G (2002) mRNA expression of the murine glycoprotein (transmembrane) nmb (Gpnmb) gene is linked to the developing retinal pigment epithelium and iris. Brain Res Gene Expr Patterns 1: 159–165. 12638126 10.1016/s1567-133x(02)00012-1

[11] Anderson MG, Smith RS, Hawes NL, Zabaleta A, Chang B, et al (2002) Mutations in genes encoding melanosomal proteins cause pigmentary glaucoma in DBA/2J mice. Nat Genet 30: 81–85. 11743578 10.1038/ng794

[12] Chi A, Valencia JC, Hu ZZ, Watabe H, Yamaguchi H, et al (2006) Proteomic and bioinformatic characterization of the biogenesis and function of melanosomes. J Proteome Res 5: 3135–3144. 17081065 10.1021/pr060363j

[13] Ripoll VM, Meadows NA, Raggatt LJ, Chang MK, Pettit AR, et al (2008) Microphthalmia transcription factor regulates the expression of the novel osteoclast factor GPNMB. Gene 413: 32–41. 18313864 10.1016/j.gene.2008.01.014

[14] Loftus SK, Antonellis A, Matera I, Renaud G, Baxter LL, et al (2009) Gpnmb is a melanoblast-expressed, MITF-dependent gene. Pigment Cell Melanoma Res 22: 99–110. 18983539 10.1111/j.1755-148X.2008.00518.x PMC2714741

[15] Saito H, Yasumoto K, Takeda K, Takahashi K, Yamamoto H, et al (2003) Microphthalmia-associated transcription factor in the Wnt signaling pathway. Pigment Cell Res 16: 261–265. 12753399 10.1034/j.1600-0749.2003.00039.x

[16] Vachtenheim J, Borovansky J (2010) “Transcription physiology” of pigment formation in melanocytes: central role of MITF. Exp Dermatol 19: 617–627. 20201954 10.1111/j.1600-0625.2009.01053.x

[17] Nishioka E, Funasaka Y, Kondoh H, Chakraborty AK, Mishima Y, et al (1999) Expression of tyrosinase, TRP-1 and TRP-2 in ultraviolet-irradiated human melanomas and melanocytes: TRP-2 protects melanoma cells from ultraviolet B induced apoptosis. Melanoma Res 9: 433–443. 10596909 10.1097/00008390-199910000-00002

[18] Mizutani Y, Hayashi N, Kawashima M, Imokawa G (2010) A single UVB exposure increases the expression of functional KIT in human melanocytes by up-regulating MITF expression through the phosphorylation of p38/CREB. Arch Dermatol Res 302: 283–294. 19937254 10.1007/s00403-009-1007-x

[19] Watabe H, Valencia JC, Yasumoto K, Kushimoto T, Ando H, et al (2004) Regulation of tyrosinase processing and trafficking by organellar pH and by proteasome activity. J Biol Chem 279: 7971–7981. 14634018 10.1074/jbc.M309714200

[20] Hoashi T, Watabe H, Muller J, Yamaguchi Y, Vieira WD, et al (2005) MART-1 is required for the function of the melanosomal matrix protein PMEL17/GP100 and the maturation of melanosomes. J Biol Chem 280: 14006–14016. 15695812 10.1074/jbc.M413692200

[21] Tadokoro T, Yamaguchi Y, Batzer J, Coelho SG, Zmudzka BZ, et al (2005) Mechanisms of skin tanning in different racial/ethnic groups in response to ultraviolet radiation. J Invest Dermatol 124: 1326–1332. 15955111 10.1111/j.0022-202X.2005.23760.x

[22] Sheng MH, Wergedal JE, Mohan S, Lau KH (2008) Osteoactivin is a novel osteoclastic protein and plays a key role in osteoclast differentiation and activity. FEBS Lett 582: 1451–1458. 18381073 10.1016/j.febslet.2008.03.030

[23] Abdelmagid SM, Barbe MF, Rico MC, Salihoglu S, Arango-Hisijara I, et al (2008) Osteoactivin, an anabolic factor that regulates osteoblast differentiation and function. Exp Cell Res 314: 2334–2351. 18555216 10.1016/j.yexcr.2008.02.006

[24] Rose AA, Annis MG, Dong Z, Pepin F, Hallett M, et al (2010) ADAM10 releases a soluble form of the GPNMB/Osteoactivin extracellular domain with angiogenic properties. PLoS One 5: e12093. 20711474 10.1371/journal.pone.0012093 PMC2919417

[25] Hoashi T, Tamaki K, Hearing VJ (2009) The secreted form of a melanocyte membrane-bound glycoprotein (Pmel17/gp100) is released by ectodomain shedding. FASEB J 24: 916–930. 19884326 10.1096/fj.09-140921 PMC2830135

[26] Le Poole IC, van den Berg FM, van den Wijngaard RM, Galloway DA, van Amstel PJ, et al (1997) Generation of a human melanocyte cell line by introduction of HPV16 E6 and E7 genes. In Vitro Cell Dev Biol Anim 33: 42–49. 9028834 10.1007/s11626-997-0021-6

[27] Haraszti T, Trantow CM, Hedberg-Buenz A, Grunze M, Anderson MG (2011) Spectral analysis by XANES reveals that GPNMB influences the chemical composition of intact melanosomes. Pigment Cell Melanoma Res 24: 187–196. 21029394 10.1111/j.1755-148X.2010.00788.x PMC3021633

[28] Owen TA, Smock SL, Prakash S, Pinder L, Brees D, et al (2003) Identification and characterization of the genes encoding human and mouse osteoactivin. Crit Rev Eukaryot Gene Expr 13: 205–220. 14696968 10.1615/critreveukaryotgeneexpr.v13.i24.130

[29] Hoek KS, Schlegel NC, Eichhoff OM, Widmer DS, Praetorius C, et al (2008) Novel MITF targets identified using a two-step DNA microarray strategy. Pigment Cell Melanoma Res 21: 665–676. 19067971 10.1111/j.1755-148X.2008.00505.x

[30] Sato S, Roberts K, Gambino G, Cook A, Kouzarides T, et al (1997) CBP/p300 as a co-factor for the Microphthalmia transcription factor. Oncogene 14: 3083–3092. 9223672 10.1038/sj.onc.1201298

[31] Jacquemin P, Lannoy VJ, O'Sullivan J, Read A, Lemaigre FP, et al (2001) The transcription factor onecut-2 controls the microphthalmia-associated transcription factor gene. Biochem Biophys Res Commun 285: 1200–1205. 11478782 10.1006/bbrc.2001.5294

[32] Grabacka M, Placha W, Urbanska K, Laidler P, Plonka PM, et al (2008) PPAR gamma regulates MITF and beta-catenin expression and promotes a differentiated phenotype in mouse melanoma S91. Pigment Cell Melanoma Res 21: 388–396. 18444964 10.1111/j.1755-148X.2008.00460.x PMC3951148

[33] Anghel SI, Rocha RC, Budinska E, Boligan KF, Abraham S, et al. (2012) Breast Cancer Suppressor Candidate-1 (BCSC-1) is a Melanoma Tumor Suppressor that Down Regulates MITF. Pigment Cell Melanoma Res. 10.1111/j.1755-148X.2012.01018.x 22594792

[34] Pierrat MJ, Marsaud V, Mauviel A, Javelaud D (2012) Expression of Microphthalmia-associated Transcription Factor (MITF), Which Is Critical for Melanoma Progression, Is Inhibited by Both Transcription Factor GLI2 and Transforming Growth Factor-beta. J Biol Chem 287: 17996–18004. 22496449 10.1074/jbc.M112.358341 PMC3365743

[35] Goodall J, Carreira S, Denat L, Kobi D, Davidson I, et al (2008) Brn-2 represses microphthalmia-associated transcription factor expression and marks a distinct subpopulation of microphthalmia-associated transcription factor-negative melanoma cells. Cancer Res 68: 7788–7794. 18829533 10.1158/0008-5472.CAN-08-1053

[36] Schepsky A, Bruser K, Gunnarsson GJ, Goodall J, Hallsson JH, et al (2006) The microphthalmia-associated transcription factor Mitf interacts with beta-catenin to determine target gene expression. Mol Cell Biol 26: 8914–8927. 17000761 10.1128/MCB.02299-05 PMC1636837

[37] Okazawa M, Shiraki T, Ninomiya H, Kobayashi S, Masaki T (1998) Endothelin-induced apoptosis of A375 human melanoma cells. J Biol Chem 273: 12584–12592. 9575219 10.1074/jbc.273.20.12584

[38] Magina S, Esteves-Pinto C, Moura E, Serrao MP, Moura D, et al (2011) Inhibition of basal and ultraviolet B-induced melanogenesis by cannabinoid CB(1) receptors: a keratinocyte-dependent effect. Arch Dermatol Res 303: 201–210. 21298280 10.1007/s00403-011-1126-z

[39] Liu Y, Ye F, Li Q, Tamiya S, Darling DS, et al (2009) Zeb1 represses Mitf and regulates pigment synthesis, cell proliferation, and epithelial morphology. Invest Ophthalmol Vis Sci 50: 5080–5088. 19515996 10.1167/iovs.08-2911 PMC3648851

[40] Livak KJ, Schmittgen TD (2001) Analysis of relative gene expression data using real-time quantitative PCR and the 2(-Delta Delta C(T)) Method. Methods 25: 402–408. 11846609 10.1006/meth.2001.1262

[41] An SM, Koh JS, Boo YC (2009) Inhibition of melanogenesis by tyrosinase siRNA in human melanocytes. BMB Reports 42: 178–183. 19336006 10.5483/bmbrep.2009.42.3.178

